# Habitual natto intake elevates serum MK-7 levels, enhances osteocalcin carboxylation, and supports bone density: a meta-analysis of Japanese evidence

**DOI:** 10.3389/fnut.2025.1713726

**Published:** 2025-11-28

**Authors:** Zhixiang Wen, Mengqi Zhen, Jing Wang, Agudamu Agudamu, Yang Zhang

**Affiliations:** 1 College of Physical Education, Hunan Normal University, Changsha, China; 2 Graduate School of Social Welfare, Sungkyunkwan University, Seoul, Republic of Korea; 3 Independent Researcher, Windermere, FL, United States

**Keywords:** bone content, bone remodeling, osteocalcin, osteoporosis, vitamin K2

## Abstract

**Objective:**

Natto, a traditional Japanese fermented soybean food, is the richest natural source of menaquinone-7 (MK-7), a form of vitamin K2 involved in osteocalcin activation and bone mineralization. This study aimed to systematically evaluate the effects of habitual natto consumption on serum MK-7 concentrations, osteocalcin carboxylation status, and bone mineral density (BMD) in Japanese populations.

**Methods:**

We searched PubMed, Web of Science, and Scopus through January 2025. Eligible studies compared habitual natto consumers with low- or non-consumers and reported outcomes for serum MK-7, carboxylated or undercarboxylated osteocalcin (OC/ucOC), or BMD. Certainty of evidence was assessed using the GRADE framework. Random-effects meta-analyses were performed using Cohen's *d* and 95% confidence interval (CI).

**Results:**

Six observational and quasi-experimental studies (*N* = 2,327) met inclusion criteria. Natto intake is associated with significantly elevated serum MK-7 [*d* = 2.10, 95% CI (1.55, 2.66) after sensitivity analysis removing an outlier], increased OC [*d* = 0.26, 95% CI (0.08, 0.43)], decreased ucOC [*d* = −0.50, 95% CI (−0.74, −0.26)], and modestly greater BMD across sites [*d* = 0.65, 95% CI (0.09, 1.21); sensitivity analysis: *d* = 0.35, 95% CI (0.21, 0.48)]. The certainty of evidence ranges from moderate for serum MK-7 to low or very low for osteocalcin and BMD outcomes, reflecting the predominance of observational designs and remaining imprecision. Publication bias appears minimal.

**Conclusions:**

Habitual natto consumption is associated with improved vitamin K status and bone metabolism markers. However, given the observational nature of the available evidence and its moderate-to-low certainty, these findings should be interpreted with caution. Natto may represent a culturally grounded dietary approach for supporting bone health and osteoporosis prevention, but its generalizability beyond Japanese populations warrants further investigation.

## 1 Introduction

Osteoporosis and age-related bone loss represent a growing global health burden, particularly in rapidly aging societies. As life expectancy increases, preserving musculoskeletal integrity becomes essential not only to reduce fracture risk but also to sustain quality of life and minimize healthcare expenditures. While pharmacologic treatments for osteoporosis are available, there is increasing interest in identifying dietary strategies that may serve as preventive or complementary approaches to maintain bone mass and reduce fracture incidence (1). Among the nutrients implicated in skeletal health, vitamin K—particularly in the form of menaquinone-7 (MK-7)—has attracted attention due to its pivotal role in the activation of bone matrix proteins and facilitation of bone mineralization.

Vitamin K is a fat-soluble compound, historically recognized for its role in the hepatic synthesis of blood coagulation proteins. However, its biological significance has since expanded to include critical functions in extrahepatic tissues (2), most notably in the skeletal system. A key role of vitamin K is to act as a cofactor for γ-glutamyl carboxylase, an enzyme responsible for the post-translational carboxylation of specific glutamic acid residues in vitamin K–dependent proteins. Among these, osteocalcin is a non-collagenous protein secreted by osteoblasts during bone formation and is the most abundant vitamin K–dependent protein in bone tissue. Structural analyses have revealed that human osteocalcin adopts a globular configuration composed of three α-helices, a hydrophobic core, an unstructured N-terminus, and an exposed C-terminus (3, 4). The three γ-carboxyglutamic acid residues critical for bone-binding functionality are located in the first helical domain at amino acid positions 17, 21, and 24. These residues require vitamin K for carboxylation (5). Once carboxylated, osteocalcin binds to calcium ions and hydroxyapatite crystals, facilitating its incorporation into the extracellular matrix of bone. In contrast, undercarboxylated osteocalcin lacks the ability to bind hydroxyapatite and remains in circulation. Elevated levels of serum undercarboxylated osteocalcin are indicative of inadequate vitamin K status and have been associated with diminished bone mineral density (BMD), impaired bone quality, and increased fracture risk, particularly in postmenopausal women and older adults (6). Notably, studies have shown that even in healthy individuals, as much as 50% of circulating osteocalcin can be undercarboxylated (7), highlighting the sensitivity of this marker to dietary vitamin K availability.

Vitamin K occurs naturally in two primary forms: phylloquinone (vitamin K1), found predominantly in leafy green vegetables, and menaquinones (vitamin K2), a group of isoprenoid derivatives synthesized by bacterial fermentation and present in certain fermented foods and animal products. The absorption efficiency of phylloquinone from plant sources is relatively low, with estimates of bioavailability ranging between 10 and 15% (8). Among the menaquinones, MK-4 and MK-7 are considered most relevant to human bone metabolism. While MK-4 is present in some animal-based foods and is also synthesized endogenously from phylloquinone, it exhibits a short half-life and limited systemic availability (9). In contrast, MK-7, characterized by a longer isoprenoid side chain, displays superior pharmacokinetics, including a markedly extended half-life and higher bioavailability, with 2.5-fold greater availability over 24 h and up to six-fold higher availability over 96 h compared to phylloquinone (10). These properties enable MK-7 to maintain more stable serum concentrations, thereby supporting sustained carboxylation of osteocalcin and other extrahepatic vitamin K–dependent proteins.

**Figure 1:**
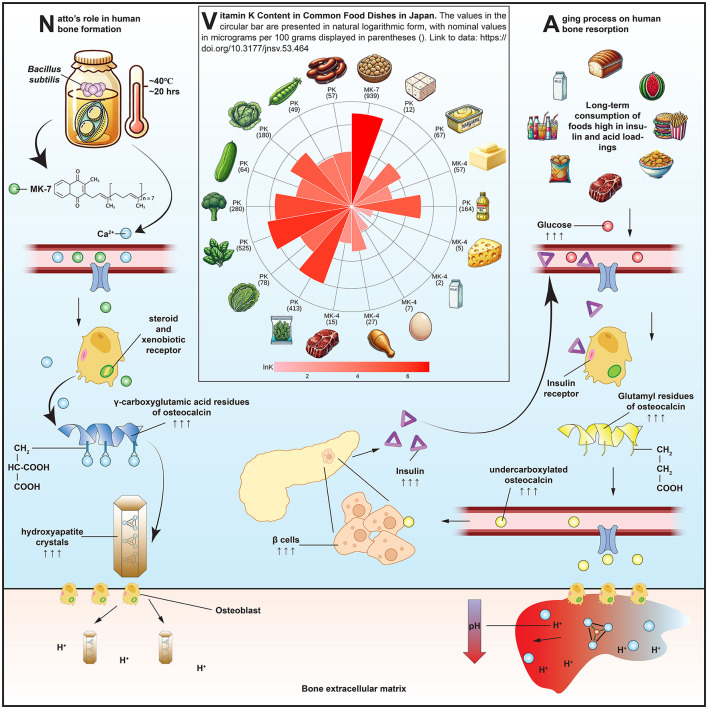
Mechanisms by which natto-derived menaquinone-7 (MK-7) supports bone health: MK-7 absorption and osteocalcin carboxylation **(left)**, vitamin K content across Japanese foods **(middle)**, and acid-induced bone resorption and systemic modulators **(right)**. Food items in the middle panel are arranged clockwise from top as follows: natto, tofu, margarine, butter, vegetable oil, cheese, whole milk, whole egg, chicken, beef, hijiki seaweed, lettuce, spinach, broccoli, cucumber, cabbage, sugar peas, and kidney beans.

Among all dietary sources of MK-7, natto—a traditional Japanese fermented soybean product produced by inoculating cooked soybeans with *Bacillus subtilis*—stands out as the most concentrated natural source. Each 50-g serving of natto typically provides approximately 380 μg of MK-7 (11), a level shown in clinical studies to elevate serum MK-7 concentrations and promote osteocalcin carboxylation (12). Given the central role of MK-7 in osteocalcin carboxylation and the unique biochemical properties of natto, it is informative to visualize how this nutrient-rich food contributes to skeletal metabolism. Figure 1 provides a conceptual overview of these mechanisms. The left panel illustrates the metabolic pathway by which MK-7—produced during the fermentation of soybeans by *Bacillus subtilis*—is absorbed and enters systemic circulation. Upon absorption, MK-7 circulates systemically and functions as a cofactor for γ-glutamyl carboxylase in osteoblasts, enabling the carboxylation of osteocalcin. This modification allows osteocalcin to bind calcium ions and integrate into hydroxyapatite crystals, thus reinforcing the structural matrix of bone. In parallel, MK-7 has been shown to act as a natural ligand for the steroid and xenobiotic receptor (13), a nuclear receptor expressed in osteoblasts. Upon activation, the steroid and xenobiotic receptor initiates transcription of genes involved in bone formation, matrix regulation, and detoxification processes, suggesting that MK-7 exerts additional genomic effects beyond its classical role in carboxylation. Importantly, natto also supplies modest amounts of dietary calcium, which serves as the critical substrate for hydroxyapatite formation and complements the action of MK-7 by providing the mineral that carboxylated osteocalcin is designed to bind. The middle panel of Figure 1 presents a radial comparison of vitamin K content across commonly consumed Japanese foods, underscoring natto's exceptional MK-7 concentration. While green leafy vegetables such as spinach and broccoli are rich in phylloquinone, their limited absorption and rapid hepatic clearance (14) reduce their efficacy in extrahepatic tissues like bone. Other fermented foods, including cheese and miso, contain various menaquinones (e.g., MK-4, MK-9), but in significantly lower quantities and with diminished bioactivity in humans. The confluence of MK-7 bioavailability, calcium content, and nuclear receptor activation positions natto as a uniquely potent functional food for skeletal health.

Despite growing scientific and consumer interest in MK-7 and bone health, the available evidence remains limited and geographically concentrated. Most clinical and epidemiological studies to date have been conducted in Japan, where natto is a cultural dietary staple. To date, no systematic review or meta-analysis has been conducted specifically on habitual natto consumption and its effects on MK-7 status and bone health outcomes. While one trial investigating MK-7 supplementation in capsule form was conducted in Norway (15), nearly all other studies have relied on Japanese cohorts and food-based MK-7 intake. This lack of comprehensive synthesis represents a critical gap in the literature and limits broader understanding of MK-7's relevance beyond regional dietary habits.

Therefore, the objective of this review is to evaluate the impact of habitual natto consumption on serum MK-7 levels, osteocalcin carboxylation status, and BMD in Japanese populations. By synthesizing evidence from observational studies and randomized controlled trials, this study aims to clarify the potential mechanistic and clinical relevance of natto as a dietary factor potentially to support bone health and mitigate age-related skeletal decline.

## 2 Methods

This systematic review and meta-analysis were conducted in accordance with the Preferred Reporting Items for Systematic Reviews and Meta-Analyses 2020 guidelines (16). A formal review protocol was not prospectively registered.

### 2.1 Eligibility criteria

Studies were considered eligible if they met predefined criteria based on the PICOS framework. The target population comprised adults residing in Japan, particularly older individuals such as postmenopausal women. The exposure of interest was habitual dietary intake of natto, assessed using validated dietary assessment tools such as food frequency questionnaires or dietary records. To maintain focus on food-based intake, studies that evaluated natto-derived MK-7 in capsule form were excluded. Comparisons were made between individuals with high vs. low or no natto intake, or across intake tertiles or quartiles reported internally by each study.

Eligible studies were required to report at least one of the following outcomes: serum MK-7 concentration, osteocalcin carboxylation status—measured as carboxylated osteocalcin or undercarboxylated osteocalcin—or BMD. Only peer-reviewed, original research articles published in English were considered for inclusion. Non-original articles, including reviews, conference proceedings, case reports, or editorials, were excluded.

### 2.2 Search strategy and study selection

A systematic search of the literature was conducted in three electronic databases: PubMed, Web of Science Core Collection, and Scopus. All records published up to January 14, 2025, were screened. The search strategy included a combination of controlled vocabulary and free-text keywords: (“natto” OR “fermented soybean”) AND (“vitamin K2” OR “menaquinone” OR “MK-7”) AND (“bone mineral density” OR “BMD” OR “osteocalcin” OR “fracture” OR “bone”). Reference lists of all included articles and relevant reviews were manually screened to identify additional eligible studies.

All identified records were imported into EndNote X21 for deduplication. Two reviewers independently screened titles and abstracts for relevance. Full texts were retrieved for studies that met initial inclusion criteria or where eligibility was unclear. Discrepancies during screening and full-text review were resolved by discussion and consensus.

### 2.3 Data extraction

Data were extracted independently by two reviewers using a standardized spreadsheet designed to capture key study characteristics and outcome measures. Extracted information included the first author's name, publication year, study design, sample size, participant demographics, methods of dietary assessment, natto intake levels, outcome measures (MK-7, carboxylated or undercarboxylated osteocalcin, and BMD), reported effect estimates, and their corresponding standard deviations, standard errors, or interquartile ranges. If essential outcome data were not available and could not be estimated, the study was excluded from the quantitative synthesis for that specific outcome. No imputation was performed for missing data or outcomes not reported.

For studies reporting multiple levels of natto consumption, only the highest intake category was extracted and compared with the reference (lowest or non-consumption group) to prevent statistical non-independence and overestimation of precision. This decision was made *a priori* to ensure consistency across studies and to emphasize the potential effect of higher habitual natto intake. When values were presented graphically, numeric data were extracted using the metaDigitise (version 1.0.1) package (17). For studies reporting medians and interquartile ranges, means and standard deviations were estimated using the metamedian (version 1.2.1) package (18). Any discrepancies between extractors were resolved through discussion until consensus was reached.

### 2.4 Risk of bias assessment

After data extraction, a formal assessment of study quality and risk of bias was undertaken to evaluate the internal validity of the included studies. Risk of bias for all included studies was assessed using the Risk of Bias in Non-randomized Studies of Interventions (ROBINS-I) tool (19). This approach was selected due to the predominance of observational and quasi-experimental designs among the included literature. Although one study incorporated an intervention with multiple intake groups, the absence of explicit randomization procedures justified the use of ROBINS-I for consistency. The tool evaluates bias across seven domains, including confounding, participant selection, exposure classification, deviations from intended interventions, missing data, outcome measurement, and selection of reported results. Two reviewers independently conducted the risk of bias assessments, with any disagreements resolved through discussion.

### 2.5 GRADE assessment

To assess the confidence in the cumulative body of evidence for each outcome, we applied the Grading of Recommendations Assessment, Development, and Evaluation (GRADE) approach (20). This method considers five domains that may reduce the certainty of evidence: risk of bias, inconsistency, indirectness, imprecision, and publication bias. The initial rating for evidence derived from observational studies was set as low but could be downgraded further or, in rare cases, upgraded based on the magnitude of effect, dose-response gradient, or if all plausible confounding would reduce an apparent effect. Each outcome was evaluated independently by two reviewers, and disagreements were resolved through consensus.

### 2.6 Data synthesis

Effect sizes were standardized using Cohen's *d*. Meta-analyses were performed using the meta (version 6.5-0) package. A random-effects model was applied to account for between-study heterogeneity. Statistical heterogeneity was quantified using the *I*^2^ statistic, with thresholds of 25%, 50%, and 75% considered to represent low, moderate, and high heterogeneity, respectively. The overall certainty of evidence for each outcome was graded using the GRADE approach, incorporating study-level risk of bias assessments and outcome-specific considerations.

### 2.7 Assessment of publication bias and sensitivity analysis

Potential publication bias was assessed using contour-enhanced funnel plots, which allow visual identification of asymmetry in relation to statistical significance thresholds. To evaluate the robustness of the meta-analytic findings, a sensitivity analysis was performed by systematically excluding potential statistical outliers and reassessing the pooled estimates.

## 3 Results

### 3.1 Study selection

**Figure 2:**
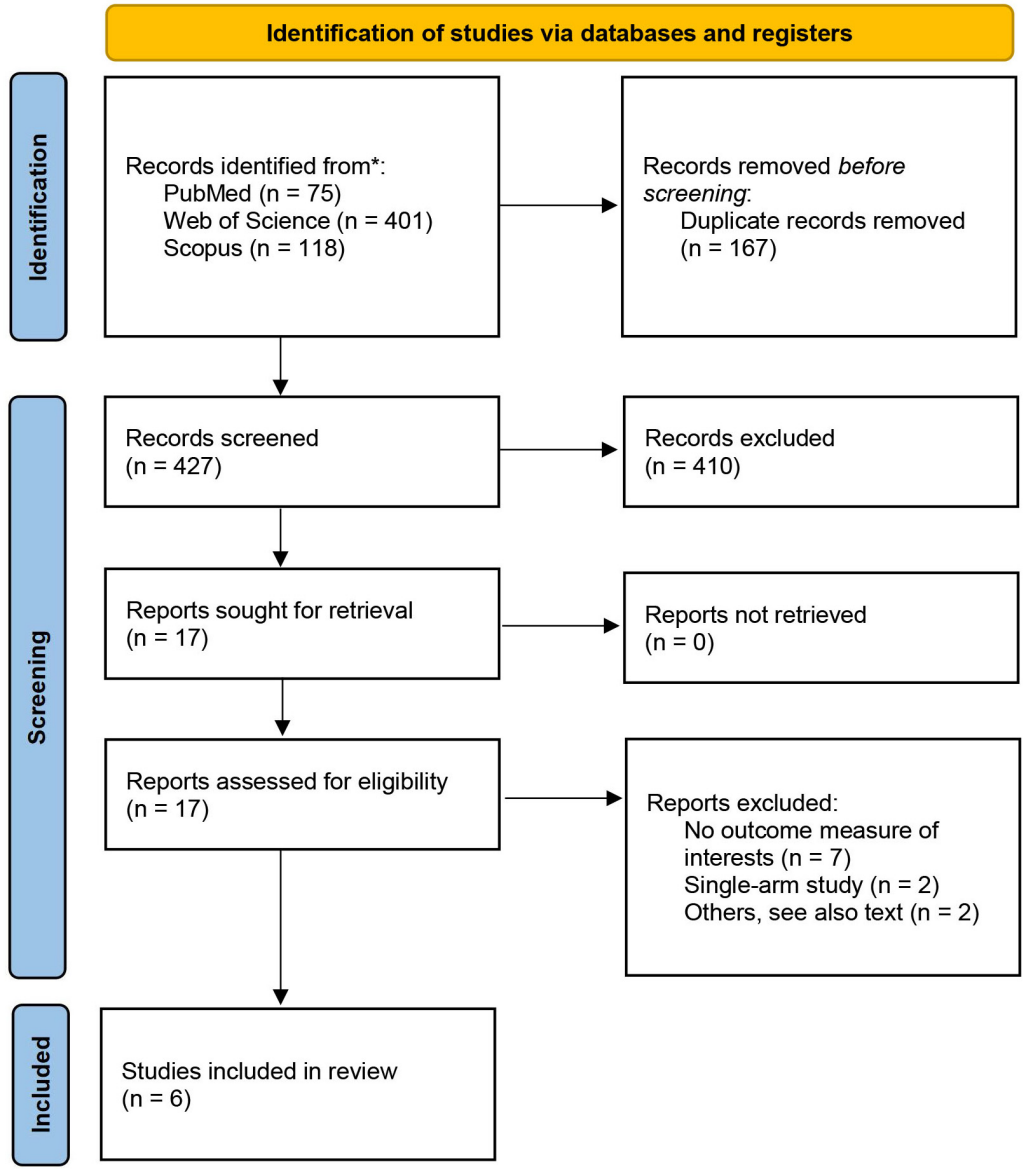
Study selection process presented as a PRISMA 2020 flow diagram.

A total of 594 records were identified through database searches, including 75 from PubMed, 401 from Web of Science, and 118 from Scopus, as shown in Figure 2. After removal of 167 duplicates, 427 unique records remained for title and abstract screening. Of these, 410 were excluded for not meeting the inclusion criteria. Seventeen full-text articles were assessed for eligibility.

Eleven studies were excluded in total, due to insufficient outcome data (*n* = 7), single-arm or uncontrolled design (*n* = 2), or other methodological limitations (*n* = 2). Two full-text articles were excluded with specific justifications. The study by Katsuyama et al. (21) was considered low quality due to ambiguous exposure classification: participants were grouped by self-reported natto intake into high, low, or no consumption categories without quantitative standardization, and the analytic design was not clearly defined. The Hirata et al. study (22), although based on the same cohort as Iwasaki et al. (23), reported overlapping participant data and was therefore excluded to avoid duplication. Additionally, only the first experiment from Tsukamoto et al. (24) was retained in the analysis. The second experiment in their study was excluded due to vague exposure definitions (“a few times per week”) and the absence of disaggregated sample sizes for each subgroup, which precluded effect size calculation. The final meta-analysis included six eligible studies (23–28).

### 3.2 Study characteristics

Study characteristics and exposure definitions are summarized in Table 1. The six included studies comprised two randomized or quasi-randomized trials and four observational studies (three cohort, one cross-sectional), all conducted in Japan between 2000 and 2021. Sample sizes ranged from 16 to over 1,000 participants. Across all included studies, data were pooled from a total of approximately 2,327 participants. Study populations were generally composed of postmenopausal women or older adults. Natto intake varied from three to seven packs per week in high-consumption groups, while control groups included non-consumers or individuals consuming fewer than one pack per week.

**Table 1:** Characteristics of included studies.

**References**	**Study design**	**Age**	**Sample size**		**Natto consumption**	
			**CON**	**IV**	**CON**	**IV**
Fujita et al. (25)	Cross sectional	≧65 years	950^*^♂	154^*^♂	<1 pack/week	≧1 pack/day
Ikeda et al. (26)	Cohort (3 years)	~65 years	210♀	81♀	No intake	>4 pack/week
Iwasaki et al. (23)	Cohort (5 years)	~64 years	56♀	104♀	Rarely	≧1 pack/day
Katsuyama et al. (28)	Randomized (1 year)	~20–50 years	18♀	18♀	No intake	3 pack/week
Kojima et al. (27)	Cohort (~15 years)	~64 years	531♀	189♀	<1 pack/week	≧7 pack/week
Tsukamoto et al. (24)	Crossover (7 days)	26–50 years	8♂	8♂	No intake	1 pack/day

CON, control (no or little natto consumption); IV, intervention (habitual natto consumption).

^*^There is a sample size difference for several measurements. This sample size indicates the overall participants in each group.

### 3.3 Risk of bias assessment

**Figure 3:**
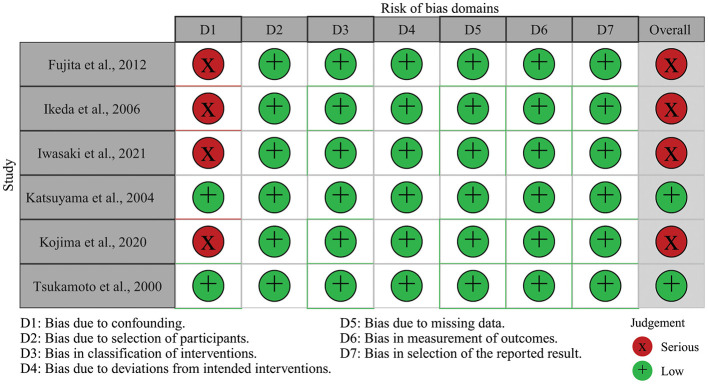
Risk of bias assessments for included studies using the ROBINS-I tool.

Risk of bias across the included studies was assessed using the ROBINS-I tool, and a visual summary of the assessment is provided in Figure 3. Most studies were judged to have a high overall risk of bias, due to serious confounding identified in the D1 domain and unclear participant-selection methods. These issues reflect inherent limitations of observational and quasi-experimental designs, particularly regarding unmeasured covariates such as diet quality, physical activity, and baseline bone status.

### 3.4 Meta-analysis results

**Figure 4:**
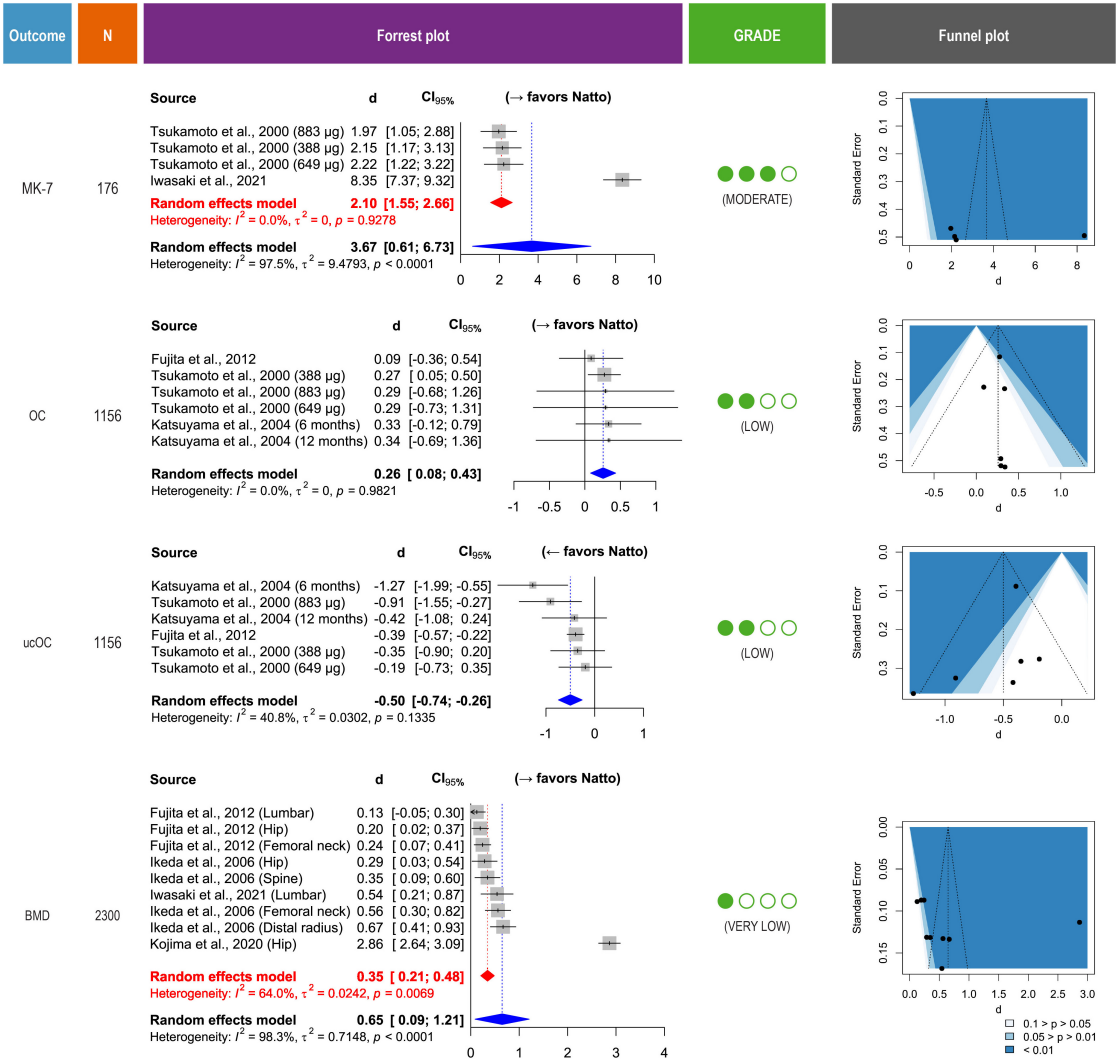
Effects of habitual natto consumption on bone health, with publication bias, and GRADE assessments. Forest plots for the effect of habitual natto consumption on serum Menaquinone-7 (MK-7), carboxylated osteocalcin (OC), undercarboxylated osteocalcin (ucOC), and bone mineral density (BMD); contour-enhanced funnel plot for assessing publication bias; and GRADE summary of evidence certainty. Effect sizes are presented as mean differences with 95% confidence intervals. Blue diamonds indicate overall pooled estimates, while red diamonds represent results of sensitivity analyses conducted for MK-7 and BMD after exclusion of outlier studies [Iwasaki et al. (23), Kojima et al. (27), respectively], which markedly reduced heterogeneity. The funnel plot includes significance contours to aid interpretation of asymmetry. Clustering of studies outside the white (non-significant) region may suggest asymmetry due to factors other than publication bias. Certainty of evidence for each outcome was rated using the GRADE framework based on risk of bias, inconsistency, indirectness, imprecision, and publication bias.

The findings from the meta-analyses are presented in Figure 4, grouped by biomarker and outcome category. Although one randomized controlled trial was included, the majority of studies were observational, which by default begin at a low-certainty rating due to potential confounding.

Four studies contributed data on the association between natto intake and circulating MK-7 levels. Pooled analysis initially indicated a large positive effect of habitual natto consumption on serum MK-7 concentration [*d* = 3.67, 95% confidence interval (0.61, 6.73)] but with high heterogeneity (*I*^2^ = 97.5%). To explore this, a sensitivity analysis excluding the Iwasaki et al. (23) cohort—identified as an outlier due to exceptionally high effect magnitude—was performed. The recalculated model yielded a pooled effect size of *d* = 2.10, 95% confidence interval [1.55, 2.66] with no residual heterogeneity (*I*^2^ = 0%), supporting a consistent association across the remaining studies. According to the GRADE assessment, the certainty of evidence for MK-7 was upgraded to moderate due to the large and precise effect size and elimination of heterogeneity.

Six comparisons from three studies were included in the analysis of serum carboxylated osteocalcin. The random-effects model indicated a modest but statistically significant increase in osteocalcin levels among habitual natto consumers compared to controls, with no observed heterogeneity. The certainty of evidence for this outcome was rated as low, reflecting the observational design of all included studies and potential confounding, though the direction of effect was consistent and biologically plausible.

Six comparisons from four studies reported undercarboxylated osteocalcin levels. Habitual natto intake was associated with a significant reduction in serum undercarboxylated osteocalcin, with moderate heterogeneity. The certainty of evidence for this outcome was rated as low, limited by residual heterogeneity and potential confounding, though supported by a coherent mechanistic rationale.

Nine comparisons from four cohort studies contributed to the BMD analysis, with measurements reported at multiple skeletal sites, including the lumbar spine, femoral neck, total hip, and distal radius. Due to variability in reporting and the limited number of studies per site, BMD outcomes were pooled across all anatomical locations to provide an overall estimate of the association between habitual natto intake and bone mass. The meta-analysis may reflect a moderate and statistically significant positive effect of natto consumption on BMD [*d* = 0.65, 95% confidence interval (0.09, 1.21)], with substantial heterogeneity (*I*^2^ = 98.3%). Notably, the Kojima et al. study (27) reported a very large effect size (*d* = 2.86). A sensitivity analysis excluding this outlier yielded a more conservative pooled estimate [*d* = 0.35, 95% confidence interval (0.21, 0.48)] with reduced heterogeneity (*I*^2^ = 64.0%). According to the GRADE framework, the certainty of evidence for BMD was downgraded to very low, reflecting the observational nature of all contributing studies, residual heterogeneity even after sensitivity analysis, and limited generalizability beyond Japanese populations.

### 3.5 Publication bias

Contour-enhanced funnel plots were generated for each outcome to assess potential publication bias (Figure 4). The plots for carboxylated osteocalcin and BMD were largely symmetric, suggesting low risk of small-study effects. For undercarboxylated osteocalcin, visual inspection revealed a relatively balanced distribution of studies across both sides of the pooled effect, further indicating minimal concern for publication bias. In contrast, the MK-7 funnel plot exhibited visible asymmetry, with an absence of studies in non-significant contour regions. However, given the consistently large effect sizes and low likelihood of a null association, this pattern may reflect a genuine underlying effect rather than selective reporting.

## 4 Discussion

This systematic review and meta-analysis is the first to synthesize Japanese evidence examining the relationship between habitual natto consumption—the richest natural source of MK-7—and bone-related outcomes, including serum MK-7, osteocalcin status, and BMD. The rationale for this study stems from a longstanding interest in food-based interventions for age-related skeletal decline. The present findings suggest an association between habitual natto consumption and indicators of vitamin K status that may relate to skeletal integrity, although direct causal links remain unproven.

Among the evaluated outcomes, the elevation of serum MK-7 levels in habitual natto consumers emerged as the most consistent. After excluding one outlier cohort with exceptionally large effects, heterogeneity was eliminated, and the pooled estimate remained large (*d* = 2.10). This strengthens confidence that the observed association reflects a genuine effect of habitual natto intake rather than a single-study artifact. Unlike other forms of vitamin K2, MK-7 derived from fermented soy has a long half-life and superior bioavailability, allowing for sustained carboxylation of vitamin K–dependent proteins in extrahepatic tissues such as bone. Thus, our analysis indicates that regular consumption of natto is associated with higher circulating MK-7 levels, consistent with its known nutrient composition, but controlled trials are needed to confirm causality.

In terms of BMD, pooled estimates from nine comparisons across four studies indicated a moderate yet statistically higher values among habitual natto consumers. These studies assessed BMD at various skeletal sites, including the lumbar spine, femoral neck, hip, and distal radius. The pooled results were therefore interpreted across anatomical locations. Sensitivity analysis excluding the outlier study markedly reduced heterogeneity (*I*^2^ = 64%) and yielded a more conservative pooled effect estimate, suggesting that study-specific factors—such as measurement site, participant age, and habitual natto intake—contributed to variance across studies. This finding aligns with broader evidence from vitamin K supplementation trials summarized by Salma et al. (29), who reported that vitamin K intake reduced fracture risk but produced smaller and less consistent effects on BMD. It is worth noting that shorter-duration intervention studies using MK-7 supplements—such as the 1-year Norwegian trial (15)—have failed to show significant BMD changes, likely due to limited exposure duration. In contrast, habitual natto intake reflects a long-term dietary behavior, potentially spanning decades, which may better capture cumulative skeletal benefits. Furthermore, BMD is only one aspect of skeletal health. Bone strength depends not only on mineral density but also on microarchitectural and material properties. Changes in crystal size and orientation, collagen integrity, and mineral composition can affect fragility independently of BMD (30). Ultrasound velocity, for instance, has emerged as a promising indicator of bone quality (31), and future trials should consider such measures alongside conventional DXA scans. Future studies may benefit from incorporating additional indicators of bone quality, such as trabecular microarchitecture or bone turnover rates, which may capture natto's effects more sensitively than BMD alone.

It is important to note that natto's influence on BMD may not be mediated by MK-7 alone. Epidemiologically, Japan has one of the lowest age-adjusted hip fracture rates among industrialized nations (32), despite its rapidly aging population. This pattern has been partly attributed to traditional dietary habits rich in soy-based foods, including natto, tofu, and miso, as well as higher fish consumption (33). These foods collectively provide varying amounts of calcium and vitamin D that may exert additive or synergistic effects on bone metabolism. Consequently, while habitual natto intake appears to enhance vitamin K status and bone turnover markers, the incremental clinical benefit in Japan—where baseline soy and fish consumption is already high—may be relatively modest. These contextual factors help explain the moderate BMD effects observed in our analysis. Further research in populations with lower background intakes of soy or fish is needed to clarify the generalizability of these findings.

From a mechanistic perspective, fermented soybeans are also rich in isoflavones—bioactive compounds with estrogen-like activity that can protect against bone loss (34). Genistein, daidzin, and glycitin—present in natto at relatively high concentrations—have been shown to promote osteoblast differentiation and inhibit osteoclastogenesis under inflammatory conditions (35). Moreover, isoflavones act as antioxidants, mitigating oxidative stress that would otherwise impair bone-forming cells (36). Importantly, natto contains isoflavone aglycones, which are absorbed more rapidly and are more bioavailable than the glycoside forms found in other soy foods. Randomized controlled trials of genistein supplementation have demonstrated improvements in femoral-neck BMD (37), lending support to this mechanistic hypothesis. Nevertheless, controlled clinical trials are needed to confirm whether MK-7 and isoflavones act synergistically in humans.

The findings related to osteocalcin status further support natto's role in bone metabolism. Habitual consumption was associated with an increase in carboxylated osteocalcin and a reduction in undercarboxylated osteocalcin, consistent with improved vitamin K status. However, the clinical interpretation of these markers is complicated by heterogeneity in assay design and antibody specificity (38, 39). Moreover, elevated undercarboxylated osteocalcin may not solely indicate inadequate vitamin K intake. Osteoclastic bone resorption also promotes decarboxylation of osteocalcin through local acidification, releasing undercarboxylated osteocalcin into circulation. This dual origin—under-carboxylation vs. active decarboxylation—makes undercarboxylated osteocalcin a sensitive but non-specific biomarker of vitamin K status. The right panel of Figure 1 conceptually illustrates this resorptive process, in which osteoclasts acidify the extracellular matrix, thereby promoting osteocalcin decarboxylation and releasing osteocalcin into the bloodstream. In addition, circulating undercarboxylated osteocalcin levels are influenced by systemic metabolic factors such as insulin and leptin (40), both of which modulate osteoblast activity and osteocalcin production. In mice, this feedback loop extends to pancreatic beta-cell proliferation and insulin secretion (41), suggesting osteocalcin's role as both a skeletal and metabolic hormone.

Broader systemic conditions may also shape the bone response to natto. The right panel of Figure 1 illustrates how dietary patterns high in acid-producing or glycotoxic foods may offset the benefits of MK-7 and osteocalcin carboxylation by promoting bone resorption through osteoclastic activation, low extracellular pH, and inflammatory mediators. Advanced glycation end products, which accumulate with age and high-sugar diets, damage collagen cross-linking, promote inflammation, and inhibit osteoblast adhesion and differentiation (42). Chronic metabolic acidosis, common in aging and protein-heavy diets, exacerbates this process by triggering osteoclastic activity to buffer systemic pH (43), leading to calcium loss, osteodystrophy, and increased fracture risk. These overlapping mechanisms underscore that while natto may improve MK-7 status and osteoblast function, its full benefit is modulated by diet quality and metabolic health.

There are several limitations to this review. Most critically, all included studies were conducted in Japanese populations, whose gut microbiota, soy metabolism, and dietary patterns may not generalize to other populations. Moreover, natto is an acquired taste, and its acceptability outside Japan may limit its public health applicability. Study designs varied, and although ROBINS-I ratings were mostly moderate, residual confounding remains a concern. Small sample sizes for some outcomes also reduced power. Finally, most studies relied on BMD rather than direct measures of bone quality or fracture incidence, limiting our ability to infer long-term clinical outcomes. Consistent with these limitations, the GRADE framework rated the overall certainty of evidence as low to moderate, despite generally consistent directions of effect across outcomes. This was primarily due to the observational nature of most included studies and limitations in precision. Given these constraints, our conclusions should be viewed as hypothesis-generating rather than confirmatory. These findings underscore the need for well-controlled, adequately powered randomized controlled trials to confirm the observed effects outside of Japan.

Despite these limitations, the present findings carry practical and clinical implications. For women, regular intake of natural natto may offer a safe, functional approach to preserving bone health, particularly during and after menopause. For men concerned about phytoestrogens, black bean natto—which contains similar MK-7 levels (11) but fewer isoflavones—may be preferable. Individuals unable or unwilling to consume natto may opt for MK-7 supplements, though they should be aware that not all MK-7 supplements are equally bioactive. Only the *trans* isomer of MK-7 has been shown to be biologically effective, while the *cis* form, often present in lower-quality formulations, is poorly absorbed and may act as a competitive inhibitor (44). Consumers and clinicians should therefore verify supplement quality and consider dietary fat co-ingestion to optimize absorption. At ~100 μg daily doses, circulating MK-7 levels can reach approximately 1.5 nM (10), approaching the upper limit of the normal range and ensuring sufficient tissue availability over 24 h. In fact, long-term evidence suggests that a daily dose of 100–200 μg MK-7 is sufficient to maintain stable circulating levels and support bone health (45). Because vitamin K interacts with anticoagulant therapy, particularly among older adults, any increase in natto or MK-7 intake should be undertaken with medical supervision to ensure safe integration into existing treatment regimens.

Clinically, these findings invite new directions for post-fracture rehabilitation. Current protocols do not routinely measure vitamin K status or MK-7 levels in osteoporotic or fracture patients. Given the mounting evidence linking osteocalcin carboxylation to bone strength and fracture risks (46, 47), there is a compelling rationale to explore natto supplementation—or MK-7 monitoring—as part of comprehensive bone recovery strategies. Future clinical trials should evaluate whether targeted MK-7 interventions can accelerate fracture healing or reduce recurrence, particularly in older adults. Incorporating MK-7 screening into standard osteoporosis workups may help identify patients who could benefit from natto-based interventions or targeted supplementation.

In conclusion, habitual natto consumption is associated with higher serum MK-7 levels, greater osteocalcin carboxylation, and modestly higher BMD. These effects appear to be mediated through complex, synergistic mechanisms involving carboxylation, antioxidant defense, hormonal signaling, and systemic acid-base balance. While further research is needed to confirm causality, assess generalizability, and establish fracture-related endpoints, natto stands out as a distinctive, culturally rooted functional food that may contribute to dietary patterns supportive of skeletal health. In a time of growing interest in food-as-medicine, reintroducing fermented foods like natto into broader global diets may not only be scientifically grounded but also a culturally respectful and sustainable tool for global bone health.

## Data Availability

Publicly available datasets were analyzed in this study. This data can be found here: Figshare (doi: 10.6084/m9.figshare.29458958.v1).
